# The University and Hospital Bulletin

**Published:** 1904-11

**Authors:** 


### The University and Hospital Bulletin

DEVOTED TO THE INTERESTS OF THE UNIVERSITY OF BUFFALO AND THE HOSPITALS.

EDITED BY
NELSON W. WILSON, M. D.

*Assistants:*
E. R. McGuire, M. D., Burton T. Simpson, M. D.

Under the auspices of the Faculty of the Medical Department of the University

M. D. Mann, A. M., M. D., Dean,
Charles G. Stockton, M. D.,
John Parmenter, M. D.,
Eli H. Long, M. D.,
Roswell Park, A. M., M. D., LL. D.,
Charles Cary, M. D.,
Herbert M. Hill, A. M., Ph. D.,
Herbert U. Williams, M. D.

AND

*Buffalo General Hospital* Henry Reed Hopkins, M. D.
*Hospital of the Sisters of Charity* Eugene A. Smith, M. D.
*German Hospital* Herman E. Hayd, M. D.

On October 28, 1904, the American Society of Clinical Surgery met in Buffalo in the surgical clinic of the General Hospital. At these society meetings no formal papers are read, the work being purely clinical and demonstrative. The arrangements of the meeting were under Dr. Roswell Park's guidance, and to the members of the society were presented by Buffalo medical men many interesting and some rare cases illustrative of surgical work in Buffalo. After the meeting Dr. Park gave a luncheon at the University Club to the society members.

---

A case which is of unusual interest was operated upon at Dr. Park's clinic during the month of October. The patient was 18 years old. He presented on the left side of his head in the parieto-frontal region a large, bulging tumor which preliminary examination showed to extend downward across the temporal region to the zygoma and apparently into the tissues of the cheek. There was a history of six years slow growth of the tumor. In operating, Dr. Park exposed both carotids, putting a provisional ligature around the right and tying off the left permanently. An incision was made from the left angle of the mouth upward to the zygoma, thence upward and forward along the long axis of the parietal tumor. The upper portion of the tumor was dissected out down to the zygoma beneath which it extended, necessitating a division of that bone. Here one portion dipped backward, another portion filling the antrum, one side of which had been destroyed; another prolongation extended into the tissues of the cheek. All the superficial portions of the tumor were removed. An effort to follow the course of what appeared to be an outgrowth of the origin of the tumor in the spheno-maxillary fossa developed a large mass which, in view of the condition of shock which was rapidly developing, it was deemed unwise to attack. The cheek wound was closed, the upper wound was packed and closed with silkworm gut sutures temporarily, the intention being to do a second operation for the removal of the balance of the tumor when the patient's condition warranted. The patient died the same night. A postmortem examination revealed the tumor origin in all probability to have been at the posterior pterygoid plate. From the antrum two masses extended each side of the posterior nares. The tumor was a fibroma and the upper portion removed from the temporo-frontal region measured relatively 150 x 100 mm.

---

A RARE condition of aneurism of the left carotid was presented at Dr. Park's clinic at the General Hospital during the month. The tumor had been of gradual growth, and when operated was the size of a mandarin orange. Dr. Park exposed both carotids, placing a provisional ligature about the right and tying off the aneurism of the left above and below the tumor after which the whole mass was excised. The object in placing the right provisional ligature was to check possible hemorrhage by way of the Circle of Willis, originating from the right internal carotid. The patient made an uninterrupted recovery.

---

DR. EUGENE A. SMITH operated for thrombosis of the left femoral artery at the Sisters' Hospital, the thrombus occluding the artery which was already thickened and hardened by arteriosclerosis.

The patient is 78 years old and five weeks before being seen by Dr. Smith had fallen down stairs and sustained fractures of three ribs on the left side. There had been some ecchymotic spots on the left lower extremity, but he did not complain of this limb particularly until five days after the accident, when he spoke of pains extending downward from the knee to the foot and into the ankle and toes. These pains were worse during the afternoon and evening, worse on rubbing the limb and exercise accentuated the discomfort; they were not relieved by any application, hot or cold, and morphine in sufficient quantity was necessary to dull the pain. When first seen, five weeks after the fall, the extremity was colder and paler than the right and moist; there was no pulsation to be felt in the femoral artery from the apex of Scarpa's triangle downward. A diagnosis of thrombosis of the femoral artery was made and amputation advised. A physical examination revealed a general condition of arteriosclerosis and heart murmurs, but the patient's general condition was good. Three weeks after examination he was seen again. In the meantime the pains had gotten worse, the second toe was dark and cold, developing odor and the patient was slightly delirious, temperature 100-101°. Amputation was urged and two days later the operation was performed after nerve blocking with cocaine by Crile's method. Owing to the heart condition ethyl chlorid was the anesthetic, three tubes being used during the operation, which consisted of amputation of the thigh at the junction of the middle and upper thirds by the circular method. Primary union followed and the patient went home in two weeks.

Postmortem of the leg showed the femoral artery to have been blocked by the thrombus from the apex of Scarpa's triangle into the popliteal space. The artery was calcified, generally speaking, but there were soft spots here and there.

---

Dr. Herbert U. Williams, professor of pathology, has returned from his vacation and renewed his work at the university. There are many improvements being made in Dr. Williams' department, and extensive research work is being done, details of which cannot, of course, at this time be made public.

---

The department of physiology, under Dr. F. C. Busch, has grown to be one of the most perfect and ably conducted in the state. In the laboratory, of which little is heard, a great deal of important work is being carried on.

---

In this year's classes there are 60 freshmen, 63 sophomores, 53 juniors, and 47 seniors. There are also a number of special students and several physicians doing post-graduate work.

---

The principal secret societies of the medical department have opened their frat. houses and are settled for the winter. Alpha chapter, Omega Upsilon Phi, is located at 393 Delaware avenue, and many of the student members have taken rooms in the house. Graduate members are invited to visit the house. A series of alumni receptions, to be held once a month, has been arranged.

---

Alpha chapter has received many letters from delegates, thanking it for its hospitality during the grand chapter convention held in Buffalo this year. Some idea of the extent of Omega

Upsilon Phi may be gained when it is known that the grand chapter convention was attended by delegates from California, Colorado, Canada, Missouri, New York, Ohio, Illinois, and Maryland.

---

THE annual alumni reception was held at the chapter house on October 8, which was attended by nearly every member on the rolls.

---

THE freshmen students were given a "smoker" on Friday evening, October 12. Many members of the faculty were present.

---

QUIZZES in all departments have begun at the chapter house. The subjects are divided among the graduate members and the work is thorough and painstaking. This year all the special subjects have been taken up and are being gone into more extensively than heretofore, and especially is this so of genitourinary work, a thorough course of which, including instrumentation, has been arranged. Other special subjects will be taken up from time to time.

---

I. C. I. SOCIETY.—The I. C. I. Society frat. house is located at 151 North Pearl street, and Mr. Louis Hengerer, the president, has issued an invitation to graduate members to inspect the society's new home.

---

QUIZZES and class instructions are under way at the frat. house, and much hard work is being done by the members. All the subjects covered each day at the university are gone over in the conferences, and the work is constantly being freshened and brought up to date. The society quiz masters are enthusiastic in their work. The I. C. I., too, is covering all the special subjects.

---

A FRESHMAN "smoker" was held during October, which was largely attended, many of the guests being members of the faculty, instructors and lecturers.

---

ALPHA OMEGA DELTA.—The A. O. D. chapter house has been located at 122 North Pearl street. It is one of the most comfortable frat. houses in Buffalo. The rooms are pretty well taken up by student members, who are all hard at work now.

The regular weekly quizzes began on October 15. The work appears to have been laid out on something like the same general plan as obtains at the other frat. houses.

The annual reception to the freshmen was held on Friday, October 8. This was the most successful freshman reception given by any of the fraternities. The entire freshman class was present, and the new students met many alumni and members of the faculty during the evening.

---

Dr. E. J. Meyer, adjunct professor of clinical surgery, and Dr. H. C. Buswell, adjunct professor of medicine, are still in Germany. Dr. Buswell is expected to return in December. Dr. Meyer has not fixed any definite date for his return.

---

Dr. L. G. Hanley, clinical professor of obstetrics, will return from his European trip in November, and resume his work at the university, giving the students the benefit of his clinics at the hospitals.

---

Dr. A. B. Wright, clinical instructor in genitourinary diseases at the dispensary of the University of Buffalo, has an article in the *New York Medical Journal* on vesical calculus, which is remarkable for the age of the patient, 7 years, the thickness of the bladder walls, 4 cm., and the duration of the trouble, 5 years.

---

The teaching staff of the medical department of the university numbers over 100, seven of whom make up the permanent faculty. There are 16 professors, adjunct professors and associate professors; 24 professors of special departments; 34 lecturers and instructors and 20 clinical instructors. There is a probability of early increase in the teaching staff by the addition of other departments and the extension of some of the special departments now existing.

Few medical schools are better equipped, or have abler teachers, than the university medical department, while as for clinical advantages Buffalo is really unexcelled with its wealth of hospitals and the almost unlimited opportunity for clinical instruction at bedside, in operating room and in dispensary and the observation of the progress of cases.

---

On October 8, Dr. Burton T. Simpson, assistant in pathology, read a paper before the Lake Erie Medical Society at Dayton, N. Y., on The microscope as an aid in diagnosis, which will be published in the next issue of the Journal.

THE first operation at the new Mercy hospital,—a case of ruptured urethra due to traumatism,—was performed by Dr. J. Henry Dowd, on October 14, 1904. Physicians present were E. M. Dooley, William G. Ring, P. H. Hourigan, James W. Nash, and Robert K. Grove.

